# The X-Linked Tumor Suppressor TSPX Interacts and Promotes Degradation of the Hepatitis B Viral Protein HBx via the Proteasome Pathway

**DOI:** 10.1371/journal.pone.0022979

**Published:** 2011-07-29

**Authors:** Tatsuo Kido, Jing-Hsiung James Ou, Yun-Fai Chris Lau

**Affiliations:** 1 Division of Cell and Developmental Genetics, Department of Medicine, Veterans Affairs Medical Center, University of California San Francisco, San Francisco, California, United States of America; 2 Department of Molecular Microbiology and Immunology, Keck School of Medicine, University of Southern California, Los Angeles, California, United States of America; Chinese University of Hong Kong, Hong Kong

## Abstract

Hepatitis B virus (HBV) infection is a major risk for hepatocellular carcinoma (HCC), and it is a serious global health problem with two billion people exposed to it worldwide. HBx, an essential factor for viral replication and a putative oncoprotein encoded by the HBV genome, has been shown to promote oncogenic properties at multiple sites in HBV-infected liver cells. The expression level of HBx closely associates with the development and progression of HCC, therefore the mechanism(s) regulating the stability of HBx is important in oncogenesis of HBV-infected cells. We demonstrate that the X-linked tumor suppressor TSPX enhances the degradation of HBx through the ubiquitin-proteasome pathway. TSPX interacts with both HBx and a proteasome 19S lid subunit RPN3 via its C-terminal acidic tail. Most importantly, over-expression of RPN3 protects HBx from, and hence acts as a negative regulator for, proteasome-dependent degradation. TSPX abrogates the RPN3-depedent stabilization of HBx, suggesting that TSPX and RPN3 act competitively in regulation of HBx stability. Since mutation and/or epigenetic repression of X-located tumor suppressor gene(s) could significantly predispose males to human cancers, our data suggest that TSPX-induced HBx degradation could play key role(s) in hepatocarcinogenesis among HBV-infected HCC patients.

## Introduction

The Y-encoded testis-specific protein Y-encoded (TSPY) and its X-chromosome homologue TSPX (also called TSPYL2, CDA1 and DENTT) are members of the SET/NAP1 superfamily of proteins, which are characterized by the presence of a highly conserved NAP-domain [1], [2], [3]. TSPY is a tandemly repeated gene mapped to the critical region of gonadoblastoma locus on the Y chromosome (GBY). It is highly expressed in gonadoblastoma, preferentially developed in XY-sex reversed patients at high frequency [4], [5], [6]. TSPY is also expressed in testicular carcinoma-in-situ and germ cell tumors and somatic cancers, including prostate cancer, melanoma, and liver cancer [7], [8], [9]. Over-expression of TSPY in cultured cells promotes cell proliferation and tumorigenicity in athymic mice [10]. TSPY interacts with cyclin B-CDK1 complex and stimulates its kinase activities and accelerates G2/M transition of the host cells [11]. It also binds the translation elongation factor eEF1A and promotes cellular protein synthesis, an essential oncogenic property of a cancer cell [12]. Hence, TSPY is considered to be a proto-oncogene on the Y chromosome. In contrast, the X-linked TSPX could function as a tumor suppressor by activating p53 [13] and inhibiting cyclin B-CDK1 activity [11]. Over-expression of TSPX retards cell cycle progression and promotes cell death [13], [14]. Hence, it is considered as a X-linked tumor suppressor. These observations suggest that, although TSPY and TSPX originated from the same ancestor gene, they play opposite roles in regulation of cell proliferation and tumorigenesis. Such contrasting properties of a pair of sex chromosome homologues raise the possibility that they might play important roles in sexual dimorphisms in certain somatic cancers, such as hepatocellular carcinoma, which significantly affects more men than women among their respective patient populations.

Human hepatitis B virus (HBV) is one of the major etiological factors for the development of hepatocellular carcinoma (HCC) [15]. Chronic carriers of HBV have a greater than 100-fold increased risk of developing HCC [16]. Approximately 350 million individuals are chronically infected with HBV worldwide, and this virus remains a global health problem with considerable morbidity and mortality, particularly among populations in Pacific Asia and central Africa [17]. HBV has a small DNA genome containing four partially overlapping open reading frames, encoding viral proteins, i.e. DNA polymerase, C, S and X proteins [18]. The HBV X protein (HBx) is essential for virus replication *in vivo* and has been postulated to be associated with initiation and progression of hepatocellular carcinoma [19], [20]. Indeed, transgenic mice expressing HBx show significant increase in incidence of HCC [21], [22]. HBx protein activates various pro-growth genes and signal transduction pathways, e.g. via CBP/p300, NF-κB, Ras/Raf/ERK pathways, and androgen receptor transactivation [18], [23]. HBV-associated hepatocarcinogenesis, however, is postulated to be complex and could have a lengthy incubation period, in which the affected hepatocytes could accumulate incremental oncogenic actions by HBx, other HBV components, and non-HBV factors, e.g. chronic liver inflammation. HBx protein is detected at high frequency in HCC patients with HBV-infection, but it is rarely detected in HBV-infected chronic hepatitis patients [24]. Hence, the stability of HBx protein is a key in the pathogenesis of HBV-mediated HCC. Normally, HBx is maintained at a very low intracellular level by proteasome-dependent degradation of the infected host cells [25]. Currently, only a few mechanisms, involving p53 and MDM2, have been proposed to regulate the HBx stability and degradation [26], [27].

In this study, we show a novel function of the X-encoded tumor suppressor TSPX in degradation of a HBV viral protein HBx. TSPX interacts with both HBx and RPN3, a subunit of the 19S proteasome lid, and enhances HBx degradation via ubiquitin-proteasome pathway. Since HBx plays crucial roles in development of hepatocellular carcinoma, our finding shed light on the functions of X-linked tumor suppressors in HBV-mediated liver cancer.

## Results

### TSPX enhances the degradation of HBx *in vivo*

**Figure 1:**
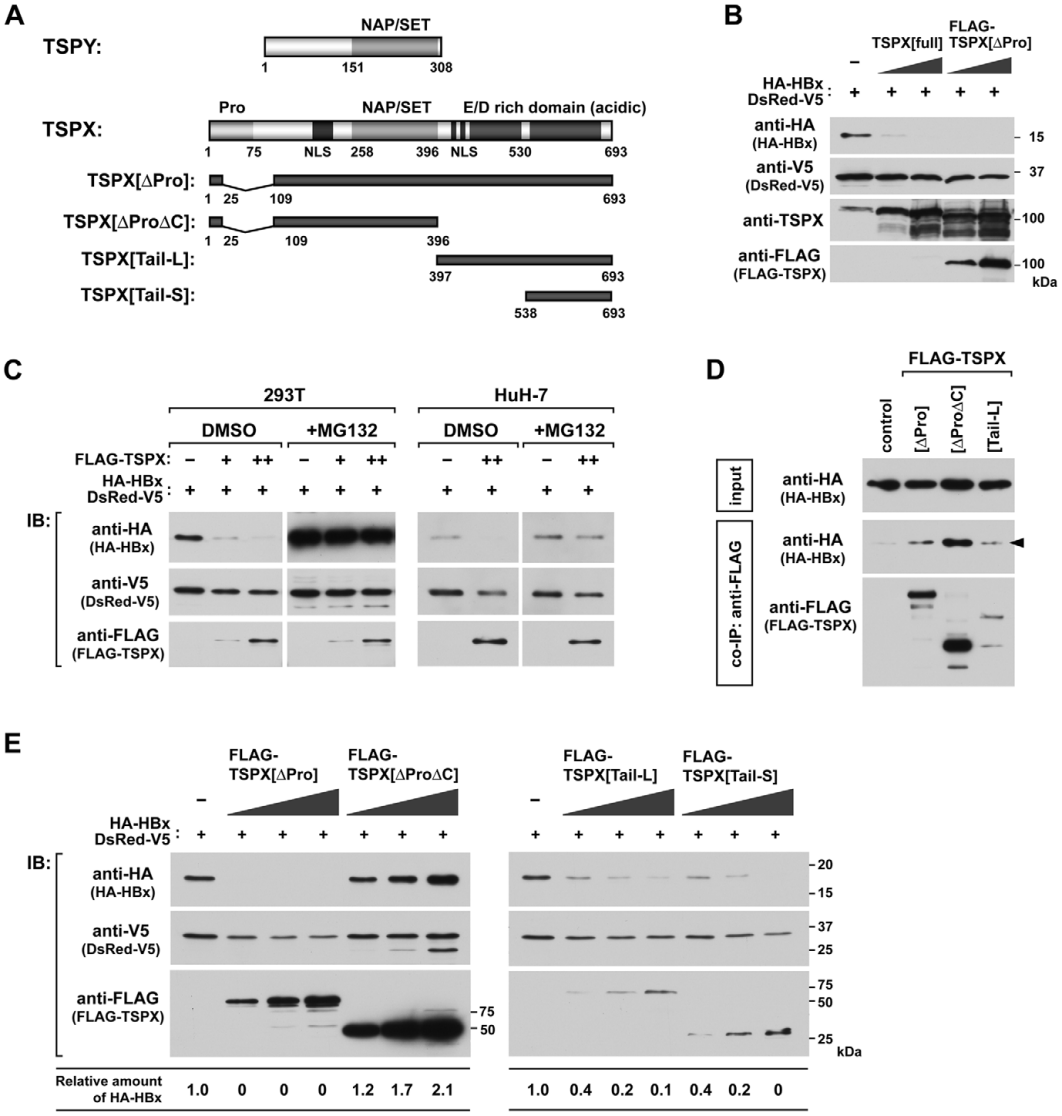
TPSX stimulates degradation of HBx in a proteasome dependent manner. (A) Structure of TSPY, TSPX, and schematic representation of the truncated mutants of TSPX used in present study. (B) Effect of co-expression with TSPX on HBx stability in mammalian cells. 293T cells were co-transfected with HA-HBx expression vector (0.2 µg/well) in the presence or absence of TSPX[full] or FLAG-TSPX[ΔPro] expression vector (0.025, 0.1 µg/well). DsRed-V5 expression vector (0.1 µg/well) was co-transfected as the internal control for monitoring the transfection efficiency. Forty-eight hours after transfection, cells were lysed and analyzed by Western-blot (immuno-blot = IB) using indicated antibodies. (C) Effect of a proteasome inhibitor MG132 on TSPX-enhanced HBx degradation. 293T cells and HuH-7 cells were co-transfected with HA-HBx expression vector (0.2 µg/well) in the presence or absence of FLAG-TSPX[ΔPro] expression vector (0.025, 0.1 µg/well). DsRed-V5 expression vector (0.1 µg/well) was co-transfected similarly as above. Twenty-four hours after transfection, cells were treated with vehicle (DMSO) or 25 µM MG132 for additional 24 h. Cells were lysed and analyzed by Western-blot using indicated antibodies. (D) Interaction of TSPX and HBx in mammalian cells. HA-HBx expression vector was co-transfected into 293T cells with expression vector of FLAG-epitope tagged TSPX mutants. Twenty-four hours after transfection, cells were treated with 20 µM MG132 for additional 24 h. Coimmunoprecipitation was performed with anti-FLAG antibody, and immunoprecipitated complexes (co-IP) were analyzed by Western blot using anti-HA and anti-FLAG antibodies. One percent of each lysate (input) was analyzed in parallel as a transfection control. (E) Mapping of the functional domain for stimulation of HBx-degradation. HA-HBx expression vector (0.1 µg/well) was co-transfected into 293T cells with FLAG-TSPX[ΔPro] (0.05, 0.1, 0.2 µg/well), FLAG-TSPX[ΔProΔC] (0.05, 0.1, 0.2 µg/well), FLAG-TSPX[Tail-L] (0.05, 0.1, 0.2 µg/well) or FLAG-TSPX[Tail-S] (0.05, 0.1, 0.2 µg/well), and analyzed as described above. The results indicate that the D/E-rich C-terminal region is sufficient to promote HBx degradation.

Since TSPY gene is frequently upregulated in HCC samples (∼50%) [8], and HBx stability and actions are closely associated with HCC development, we had examined the probable functions of TSPY and its X-linked homologue TSPX in HBV-mediated HCC. To address this problem, we first investigated the effect of TSPY and TSPX on the expression levels of HBx in transiently transfected cells. Hemagglutinin (HA) epitope-tagged HBx (HA-HBx) expression vector [28] was co-transfected with full-length TSPX (TSPX[full]), FLAG-tagged TSPX[Δ26–108] (FLAG-TSPX[ΔPro]) or TSPY expression plasmids into 293T cells. Our results showed that when HA-HBx was co-transfected with TSPX, the levels of HA-HBx protein were significantly repressed (Figure 1B) while no significant change was observed in the cells co-transfected with TSPY expression vectors (Figure S1). Since FLAG-TSPX[ΔPro] also enhanced HBx degradation as well as full-length TSPX, the proline-rich domain of TSPX may not function in HBx-degradation. The same result was obtained from the experiment using human hepatocellular carcinoma cell line HuH-7 cells (Figure 1C). Treatment with the proteasome inhibitor MG132 dramatically increased HA-HBx, and abolished the effect of FLAG-TSPX[ΔPro] in both 293T and HuH-7 cells (Figure 1C). These results suggest that TSPX down regulates HBx protein in a proteasome-dependent manner. The differential functions of TSPX and TSPY are quite interesting since this pair of homologues were initially evolved from the same ancestral gene. Although TSPX and TSPY share a highly conserved SET/NAP domain, TSPX harbors a N-terminal proline-rich domain and a C-terminal aspartic acid/glutamic acid (D/E)-rich domain, which are absent from TSPY (Figure 1A).

Co-immunoprecipitation (co-IP) assays demonstrated that HA-HBx could be co-immunoprecipitated with TSPX variants with the deleted N-terminal proline-rich domain (FLAG-TSPX[ΔPro]), deleted both N-terminal and carboxyl domains (FLAG-TSPX[ΔProΔC]) or with the C-terminal acidic domain alone (FLAG-TSPX[Tail-L]) (Figure 1D). These results suggests that TSPX interacts with HBx via multiple sites, including SET/NAP and C-terminal acidic domains, and may either directly or indirectly associate with its promoting activities for HBx degradation.

To determine the probable domain of TSPX involved in its promotion of HBx degradation, variant FLAG-tagged TSPX expression vectors harboring deletions of one or two of TSPX domains were co-transfected with HA-HBx into 293T cells. Our results showed that, while deletion of the C-terminal acidic domain alone or in combination with the proline-rich domain (TSPX[ΔProΔC]) abolished the TSPX function promoting HBx degradation while deletion of the N-terminal proline-rich domain alone did not, suggesting that the C-terminal acidic domain is important for its function in HBx degradation. Significantly, the variant TSPX constructs expressing either the complete (TSPX[Tail-L]) or an abbreviated version (TSPX[Tail-S]) of its C-terminal acidic domain were capable of promoting the degradation of the HA-HBx protein (Figure 1E). These data suggest that the mediator domain for HBx degradation is located within the D/E-rich C-terminal domain (amino acid residues 538–693) of TSPX.

**Figure 2:**
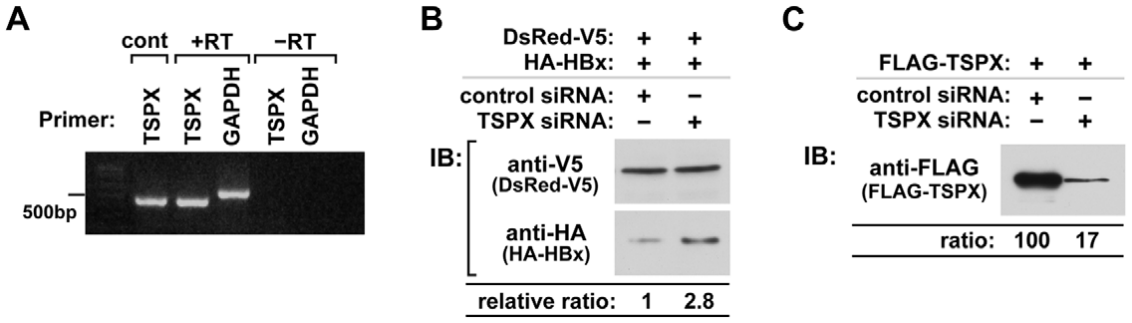
Endogenously expressed TSPX enhances HBx degradation in 293T cells. (A) 293T cells express endogenous TSPX. Total RNA isolated from 293T cells were analyzed by RT-PCR with primer pairs for TSPX[538–693] and GAPDH using a standard technique. Control, PCR product with p3×FLAG-TSPX[ΔPro]; +RT, with reverse transcriptase; -RT, without reverse transcriptase. (B) Repression of endogenous TSPX increased the expression of HA-HBx in 293T cells. HA-HBx expression vector (50 ng/well) and DsRed-V5 expression vector (50 ng/well) were co-transfected into 293T cells with either control siRNA (4 pmol/well) or TSPX siRNA (4 pmol/well). Forty-eight hours after transfection, cells were lysed and analyzed by Western-blot using anti-V5 and anti-HA antibodies. Treatment with TSPX siRNA resulted in significant increase in HA-HBx expression (2.8 fold). (C) Effect of TSPX siRNA on TSPX expression. FLAG-TSPX[ΔPro] expression vector (0.1 µg/well) was co-transfected into 293T cells with control siRNA (4 pmol/well) or TSPX siRNA (4 pmol/well). Forty-eight hours after transfection, cells were lysed and analyzed by western-blot using anti-FLAG antibody. TSPX siRNA significantly decreased the protein level of FLAG-TSPX[ΔPro].

Expression analysis using reverse transcription polymerase chain reaction (RT-PCR), demonstrated that 293T cells expressed the endogenous TSPX (Figure 2A). To determine whether endogenously expressed TSPX could also enhance the HBx degradation, we next knocked down the endogenous TSPX by using small interfering RNA (siRNA). In the cells co-transfected with TSPX siRNA, the expression level of HA-HBx was significantly increased (2.8 folds), whereas the expression level of DsRed-V5 was not affected (Figure 2B). Further, co-transfection of TSPX siRNA also significantly decreased the expression level of an exogenously transfected FLAG-TSPX[ΔPro] (Figure 2C). These observations support the hypothesis that endogenous or exogenously transfected TSPX plays critical roles in HBx degradation.

### TSPX enhances HBx degradation via ubiquitin-proteasome pathway

**Figure 3:**
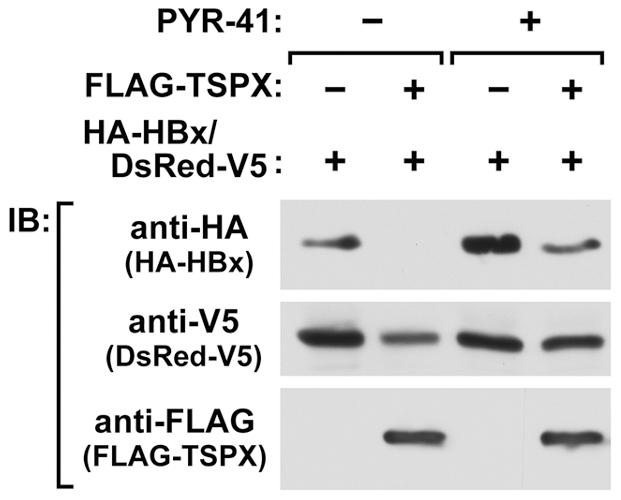
TSPX mediates HBx degradation through ubiquitin-proteasome pathway. Ubiquitin-activating enzyme inhibitor PYR-41 inhibits TSPX-mediated enhancement of HBx-degradation. HA-HBx expression vector (0.2 µg/well) and DsRed-V5 expression vector (0.1 µg/well) were co-transfected into 293T cells with FLAG-TSPX[ΔPro] (0.05 µg/well). Twenty-four hours after transfection, cells were treated with either vehicle (DMSO) or 50 µM PYR-41 for additional 24 h. Cell lysates were analyzed by Western blot using anti-HA, anti-V5 and anti-FLAG antibodies. Treatment with PYR-41 significantly increased the HA-HBx even in the presence of TSPX.

Previous studies suggest that HBx is degraded through both ubiquitin-dependent and -independent proteasome pathways [29]. Ubiquitylation is processed sequentially by ubiquitin-activating enzyme (E1), ubiquitin-conjugating enzyme (E2) and ubiquitin protein ligase (E3). In mammalian cells, while only a few types of E1 enzymes are essential, more than 500 distinct ubiquitin E3 ligases confer substrate specificity for ubiquitylation [30]. In order to determine whether TSPX enhances the HBx degradation through the ubiquitin-dependent pathway, we used PYR-41, an E1 inhibitor that blocks the activation and subsequent transfer of ubiquitin to substrate [31]. PYR-41 treatment greatly increased the levels of HA-HBx in the cells, in the presence of a co-transfected TSPX expression vector (Figure 3), suggesting that TSPX promotes HBx degradation via the ubiquitin-dependent proteasome pathway.

### TSPX interacts with a proteasome component RPN3 and inhibits the protective function of RPN3 on HBx

**Figure 4:**
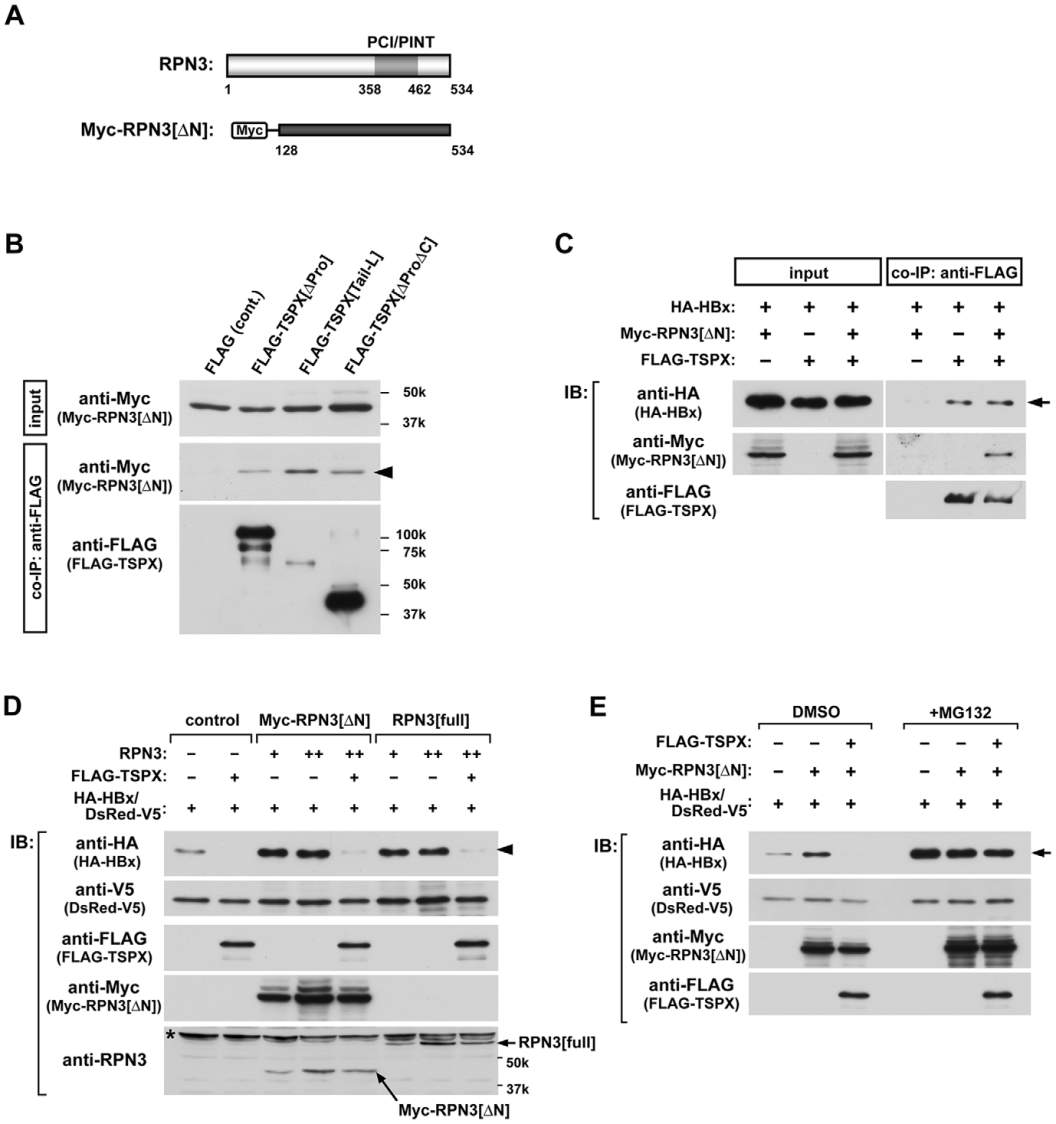
TSPX interacts with RPN3, and abrogates the protective effect of RPN3 on HBx-degradation. (A) Structure of RPN3 and schematic representation of the truncated mutant of RPN3. The position of PCI/PINT domain is as marked. N-terminal truncated RPN3 (residues 128–534 a.a., termed as RPN3[ΔN]) was cloned into pCMV-Myc vector to express a Myc epitope-tagged product. (B) Interaction of TSPX and RPN3 in mammalian cells. Myc-RPN3[ΔN] was co-transfected with p3×FLAG-CMV7 (FLAG control), FLAG-TSPX[ΔPro], FLAG-TSPX[Tail-L] or FLAG-TSPX[ΔProΔC] into 293T cells. Immunoprecipitations were carried out as before using anti-FLAG antibody. Immunoblots were probed with anti-Myc and anti-FLAG antibodies. The immunoblots indicate that human RPN3 co-immunoprecipitates with human TSPX. (C) RPN3 did not interfere the interaction between TSPX and HBx. HA-HBx and FLAG-TSPX[ΔPro] expression vectors were co-transfected with or without Myc-RPN3[ΔN] vector into 293T cells. Immunoprecipitations were carried out using anti-FLAG antibody. Immunoblots were probed with anti-HA, anti-Myc and anti-FLAG antibodies, respectively. The results indicate that interaction between HA-HBx and FLAG-TSPX was not affected by co-expression of Myc-RPN3[ΔN]. (D) Over-expression of RPN3 protected HBx from protein-degradation, and TSPX overcomes the protective effect of RPN3. 293T cells were co-transfected with HA-HBx (0.2 µg/well), Myc-RPN[ΔN] (0.05, 0.1 µg/well), RPN3[full] (0.05, 0.1 µg/well) and/or FLAG-TSPX[ΔPro] (0.05 µg/well) as indicated in the figure. pcDNA-DsRed-V5 expression vector (0.1 µg/well) was co-transfected as the internal control for monitoring the transfection efficiency. Cells were lysed 48 h after transfection, and analyzed by Western blot using indicated antibodies. Although co-transfection of RPN3[full] or RPN3[ΔN] resulted in the increase of HA-HBx, TSPX significantly decreased the level of HBx even in the presence of RPN3. (E) Over-expression of Myc-RPN3[ΔN] did not affect on the transcription of HA-HBx. 293T cells were co-transfected with HA-HBx (0.2 µg/well), Myc-RPN[ΔN] (0.1 µg/well), and/or FLAG-TSPX[ΔPro] (0.05 µg/well) as indicated in figure. pcDNA-DsRed-V5 (0.1 µg/well) was co-transfected as the internal control for monitoring the transfection efficiency. Twenty-four hours after transfection, cells were treated with either vehicle (DMSO) or 25 µM MG132 for additional 24 h. Cells were lysed and analyzed by Western blot using anti-HA, anti-V5, anti-Myc, and anti-FLAG antibodies. Co-transfection of Myc-RPN3[ΔN] increased HA-HBx (DMSO). No significant difference was observed in HA-HBx level in the cells treated with MG132 (+MG132).

Although we have demonstrated that TSPX promotes HBx degradation via its C-terminal D/E-rich domain by the ubiquitin-dependent proteasome pathway, the exact mechanism is still uncertain. This domain bears little amino acid homology to other proteins, except the Deleted in Split hand/Split foot 1 (DSS1, also called Sem1), which also harbors a D/E-rich domain. Noteworthy, DSS1 mediates various protein-protein interactions including BRCA2-RAD51 [32], and is also essential for the maintenance of 19S proteasome cap particle [33], which is required for recognition and regulation of degradation of ubiquitinated protein [34]. The 19S, in combination with the 20S proteolytic core particle, forms the 26S proteasome [34]. Further, numerous accessory proteins, including proteasome assembly chaperones and adaptor proteins that deliver the substrates to proteasome, play important roles to regulate the efficiency of critical steps in proteasome biogenesis [35]. DSS1 is known to interact with the RPN3 (also called as PSMD3 and S3) subunit of the 19S regulatory particle through its D/E-rich acidic region [36]. RPN3 harbors a PCI-domain that is characteristic for subunits of proteasome lid, COP9 signalosome (CSN) and eIF3 complex [32]. These observations suggest that the D/E-rich C-terminal domain of TSPX could interact with RPN3 and play a role in HBx degradation. To test this postulation, we had investigated the interactions between RPN3 and TSPX and its different variants in transfected cells. Co-IP assay using Myc-tagged RPN3[ΔN], bearing a PCI-domain (Figure 4A), demonstrated that Myc-RPN3[ΔN] was co-immunoprecipitated with FLAG-TSPX[Tail-L] mutant (Figure 4B). Unexpectedly, FLAG-TSPX[ΔProΔC] also interacted with RPN3, suggesting that TSPX interacts with RPN3 through multiple sites. In addition, co-IP assays demonstrated that interaction between FLAG-TSPX[ΔPro] and HA-HBx was not competitively inhibited by Myc-RPN3[ΔN] (Figure 4C).

**Figure 5:**
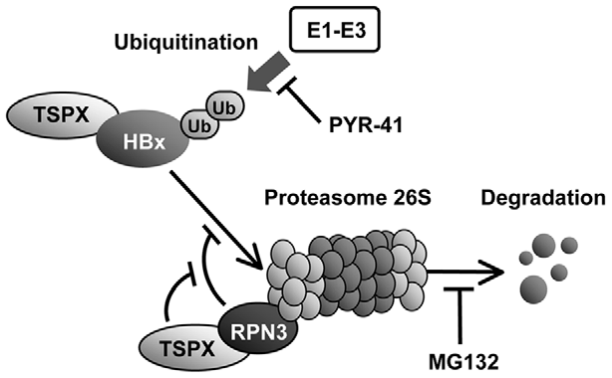
A model illustrating the potential roles of TSPX and RPN3 as regulators of ubiquitin-proteasome dependent HBx degradation. TSPX enhances the HBx degradation by recruiting HBx to proteasome complex, and inhibiting the protective function of PRN3 on HBx-degradation. E1-E3 indicates the ubiquitylation cascade including E1, E2, and E3 enzymes. MG132-responsive and PYR-41-responsive sites are also indicated.

Next, we investigated the effect of TSPX on the RPN3-dependent regulation of HBx-degradation. Co-transfection with either Myc-RPN3[ΔN] or full-length RPN3 expression vector significantly increased HA-HBx, and over-expression of FLAG-TSPX[ΔPro] abolished the effect of RPN3 on HBx-degradation (Figure 4D). However, in the cells treated with MG132, co-expression of Myc-RPN3[ΔN] did not affected the level of HA-HBx (Figure 4E), suggesting that RPN3 protects HBx from proteasome-dependent degradation. Taken together, TSPX could interact with and inhibit the protective function of RPN3 on HBx, thereby promoting the proteosomal degradation of HBx (Figure 5).

## Discussion

In the present study, we show, for the first time, that the X-linked tumor suppressor TSPX enhances HBx-degradation by inhibiting a proteasome regulatory subunit RPN3. Whereas a couple of mechanisms including p53 and MDM2 are suggested to stimulate HBx-degradation [26], [27], our data represents a novel pathway for HBx-degradation. Currently, we are uncertain how important is each of these HBx degradation pathways in modulating HBx abundance and oncogenic functions in HCC development. Significantly, the effective domain responsible for promoting HBx degradation in TSPX is mapped to the D/E-rich C-terminal domain, which also participates in the negative regulation of cyclin-B/CDK1 kinase activity [11] and retardation of cell cycle progression [37]. The Y-located TSPY, on the other hand, lacks the D/E-rich C-terminal domain, and functions as an oncoprotein promoting cyclin B-CDK1 kinase activities and cell proliferation [10], [11]. The present study further supports the hypothesis that TSPX is a tumor suppressor and exerts its anti-tumorigenic functions via its D/E-rich C-terminal domain.

Epidemiological studies demonstrated that males are at significantly high risk in developing HBV-related HCC, with a male to female ratio as high as 7∶1 as compared to that (2–3∶1, male to female) associated with hepatitis C virus (HCV)-related HCC [38], [39]. Such gender disparity has been attributed to hormonal regulation of HBx expression in a positive feedback loop [23], [40]. HBx has been shown to promote androgen receptor transactivation, while its expression is enhanced by androgen receptor via a couple of androgen response elements located within the enhancer region of HBV-genome [23]. Our study, however, shows that an X-linked tumor suppressor is capable of promoting HBx degradation. TSPX is ubiquitously expressed in normal tissues including liver [3], but its expression is markedly down regulated in primary tumors and human tumor cell lines [14]. Since male has one X chromosome, any loss-of-function mutation(s) and/or epigenetic dysregulation of X-linked tumor suppressors could render males without the corresponding oncogenic protection, thereby promoting carcinogenesis in male-preferential manner(s) [41]. Hence, genetic/epigenetic inactivation of TSPX gene could closely associate with the HBV-mediated HCC development. Further, since pro-oncogenic TSPY expression has been documented in selected HCC specimens [8], it could exert a male-specific effect(s) on the overall complex etiology for HBV-associated HCC in addition to inactivation mutation of its X-homologue, TSPX. Further studies on the roles of TSPX and TSPY in hepatocarcinogenesis could shed critical insights on the HBV-mediated pathologic process(es), and could lead to developments of genderized strategies for the prevention, diagnosis and treatments of HBV-associated liver cancer.

## Materials and Methods

### Plasmids

p3×FLAG-TSPX[ΔPro] and p3×FLAG-TSPX[ΔProΔC] were described previously [11]. TSPX[Tail-L], TSPX[Tail-S], and DsRed2 were generated by standard PCR amplification and insertion into respective expression plasmids, p3×FLAG-CMV7 (Sigma-Aldrich, St. Louis, MO) or pcDNA3.1-V5/His (Invitrogen, Carlsbad, CA), to express FLAG epitope-tagged or V5 epitope-tagged products respectively. Primers used in PCR cloning were; TSPX-397F, 5′-GCC TCG AGA GAG AAA GGG GCT CCA GGA TAA AG-3′; TSPX-538F, 5′-GCC TCG AGA CTT ACG GCA ACA ACT TCT TCA AA-3′; TSPX-693R, 5′-GCG TCG ACT TAT CCG GTT TTC CCC CTC TTC CC-3′; DsRed-F, 5′-GCG GAT CCA TGG CCT CCT CCG AGA ACG T-3′; DsRed-R, 5′-GCG GAT CCC TAG AAT TCC AGG AAC AGG TGG TGG CGG C-3′. HA-HBx expression plasmid pHAX was generated as described previously [28]. Full-length RPN3 expression vector, pCMV-SPORT6-RPN3, was purchased from Open Biosystmens (Huntsville, AL). The DNA fragment encoding RPN3[residues 128–534a.a.] was excised from pCMV-SPORT6-RPN3 using *Xho*I, and inserted into *Xho*I site of pCMV-Myc (Clontech/Takara bio, Mountain View, CA), resulting in pCMV-Myc-RPN3[ΔN].

### Cell culture

293T cells and HuH7 cells were obtained from the American Type Culture Collection (Manassas, VA) and were maintained in Dulbecco's modified Eagle medium (DMEM) supplemented with 10% fetal bovine serum (Sigma-Aldrich). One day before transfection, 293T cells were plated in 24 well plates at a density of 1.0×10^5^ cells/well, and HuH-7 cells were plated at 2×10^4^ cells/well. The cells were transfected with the above-mentioned plasmids as indicated in figure legends, using FuGENE6 (Roche Applied Science, Indianapolis, IN). Respective empty plasmids were added to the DNA mixture to ensure that equal amount of DNA was transfected in each sample. Forty-eight hours post-transfection, cells were lysed in 100 µL of SDS-PAGE sample buffer and subjected to Western-blot analysis. For HBx degradation assays, cells were treated with 25 µM MG132 (Calbiochem/EMD Chemicals, Gibbstown, NJ) or 50 µM PYR-41 (Calbiochem/EMD Chemicals) for 24 h before cell lysis. DMSO was used as vehicle.

### siRNA transfection

The siRNA targeted against TSPX and non-targeting control were purchased from Ambion/Applied Biosystems (Austin, TX). 293T cells plated in 24 well plate were co-transfected with siRNA (4 pmol/well), HA-HBx expression vector (50 ng/well) and DsRed2-V5 expression vector (50 ng/well) using HiPerFect siRNA transfection reagent (QIAGEN, Valencia, CA). Forty-eight hours after transfection, cells were lysed and analyzed by Western-blot.

### Western blot

Western-blot analysis was performed as described previously [12], using anti-FLAG rabbit IgG (Sigma-Aldrich), anti-FLAG mouse IgG (Sigma-Aldrich), anti-HA rabbit IgG (Clontech/Takara bio), anti-Myc mouse IgG (Santa Cruz Biotechnology, Santa Cruz, CA), anti-Myc rabbit IgG (Upstate/Millipore, Charlottesville, VA), anti-RPN3/PSMD3 antibody (Sigma-Aldrich), anti-TSPX/TSPYL2 rabbit antibody (Proteintech, Chicago, IL), anti-V5 rabbit IgG (Abcam, Cambridge, MA), and anti-V5 mouse IgG (Abcam).

### Co-immunoprecipitation assay

293T cells were transfected with the above-mentioned constructs using FuGENE6 (Roche Applied Science, Indianapolis, IN). Respective empty plasmids were added to the DNA mixture to ensure that equal amount of DNA was transfected in each sample. Two days post-transfection, the cells were lysed in co-IP buffer (20 mM Tris-HCl [pH 7.2], 300 mM NaCl, 20% glycerol, 1% NP-40), and incubated with anti-FLAG antibody conjugated on agarose beads (EZview Red anti-FLAG M2 affinity Gel, Sigma-Aldrich) at 4°C overnight as described previously [12]. The immunoprecipitate was washed with co-IP buffer for 1 hr at 4°C, and subjected to Western-blot analysis. In co-immunoprecipitation assays for the interactions with HA-HBx, cells were treated with 20 µM MG132 for 24 h before cell lysis.

### Reverse transcription-polymerase chain reaction (RT-PCR)

Total cellular RNA was isolated from 293T cells using RNeasy Mini kit (QIAGEN) according to vendor's instructions. First-strand cDNA was synthesized using Superscript III (Invitrogen). PCR was performed using specific primers; TSPX-397F and TSPX-693R for TSPX; hGAPDH-F (5′-CCA CCC ATG GCA AAT TCC ATG GCA-3′) and hGAPDH-R (5′-TCT AGA CGG CAG GTC AGG TCC ACC-3′) for GAPDH. Denaturation was for 1 min at 94°C, annealing for 1 min at 50–60°C, and synthesis for 1 min at 72°C.

## Supporting Information

Figure S1 **The D/E-rich C-terminal region of TSPX is critical for enhancing HBx-degradation.** 293T cells were co-transfected with HA-HBx expression vector (0.2 µg/well) and FLAG-TSPX[ΔPro], FLAG-TSPX[residues 112–693] (FLAG-TSPX[ΔN]), FLAG-TSPX[ΔProΔC] or FLAG-TSPY expression vector (0.3 µg/well) as indicated. Two days after transfection, cells were lysed and analyzed by Western blot using anti-HA, anti-FLAG, and anti-βactin antibodies. FLAG-TSPX[ΔPro] and FLAG-TSPX[ΔN] significantly down-regulated HA-HBx, while FLAG-TSPX[ΔProΔC] and FLAG-TSPY did not.
(TIF)
