# Supplementary figures and images for: The X-Linked Tumor Suppressor TSPX Interacts and Promotes Degradation of the Hepatitis B Viral Protein HBx via the Proteasome Pathway
Source: PLoS One. 2011 Jul 29;6(7):e22979. doi: 10.1371/journal.pone.0022979 (PMC3146538; doi:10.1371/journal.pone.0022979)

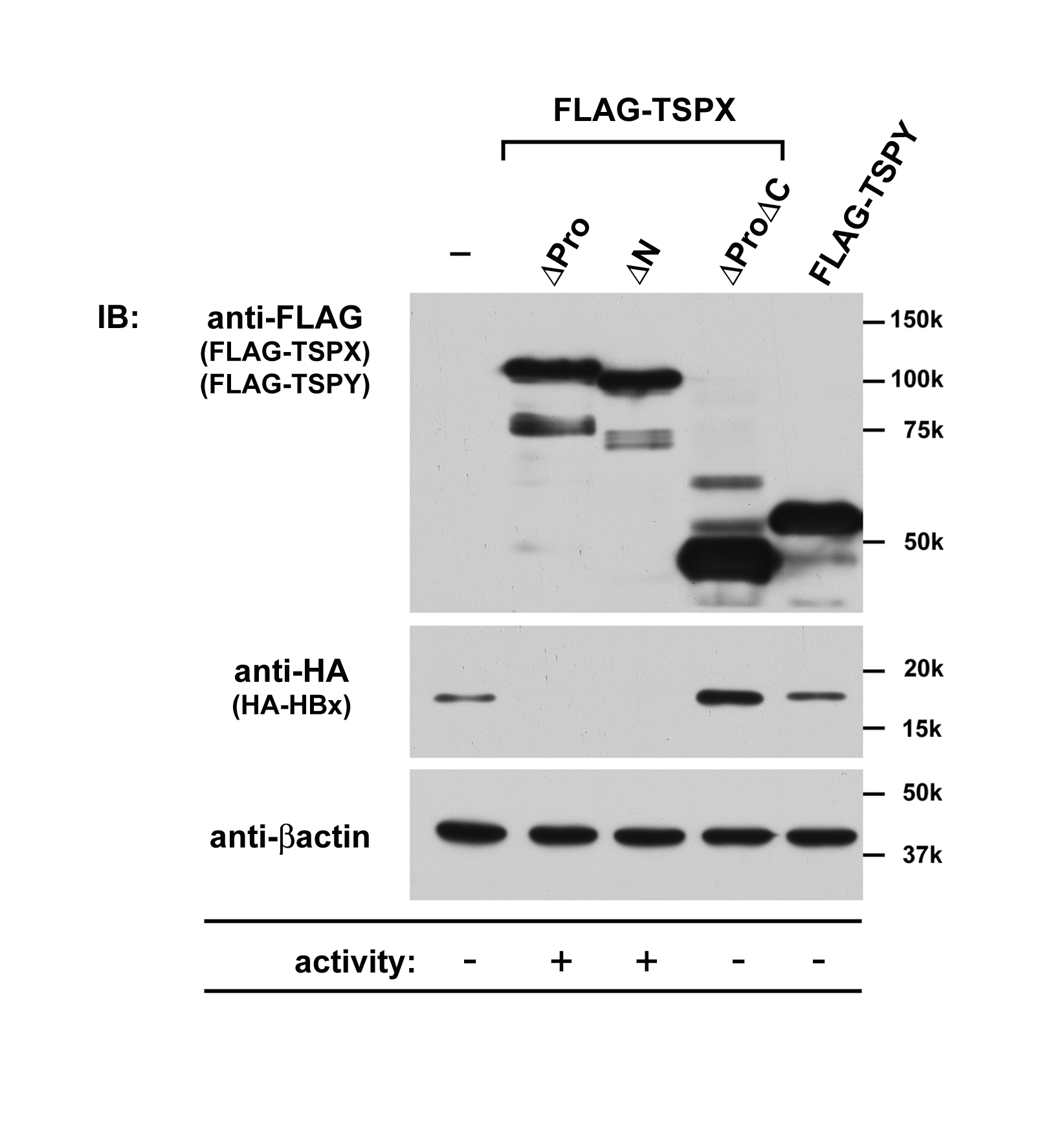

Supplement: Figure S1 — The D/E-rich C-terminal region of TSPX is critical for enhancing HBx-degradation. 293T cells were co-transfected with HA-HBx expression vector (0.2 µg/well) and FLAG-TSPX[ΔPro], FLAG-TSPX[residues 112–693] (FLAG-TSPX[ΔN]), FLAG-TSPX[ΔProΔC] or FLAG-TSPY expression vector (0.3 µg/well) as indicated. Two days after transfection, cells were lysed and analyzed by Western blot using anti-HA, anti-FLAG, and anti-βactin antibodies. FLAG-TSPX[ΔPro] and FLAG-TSPX[ΔN] significantly down-regulated HA-HBx, while FLAG-TSPX[ΔProΔC] and FLAG-TSPY did not. (TIF) [file pone.0022979.s001.tif]
